# Chorea Associated with Lamotrigine Use

**DOI:** 10.5334/tohm.751

**Published:** 2023-02-20

**Authors:** Sofia Rael, Negin Badihian, Kelsey M. Smith, Elizabeth A. Coon

**Affiliations:** 1 Mayo Clinic Alix School of Medicine, Rochester, MN; 2 Department of Neurology, Mayo Clinic, Rochester, MN

**Keywords:** Chorea, hyperkinetic, movement disorder, lamotrigine, depression, antiseizure medications

## Abstract

**Background:**

Movement disorders, including chorea, have been cited as a side effect of lamotrigine use. However, the association is controversial and clinical characteristics in such cases are unclear. We sought to explore whether chorea may be associated with lamotrigine use.

**Methods:**

We performed a retrospective chart review of all patients diagnosed with chorea who had concurrent use of lamotrigine between 2000–2022. Demographic information and clinical characteristics were analyzed, including medical comorbidities and concurrent medication use. A literature search and review were conducted, with additional cases of lamotrigine-associated chorea analyzed.

**Results:**

Eight patients met the inclusion criteria for the retrospective review. In 7 patients, other causes of chorea were considered more likely. However, a 58-year-old woman with bipolar disorder on lamotrigine for mood stabilization had a clear association of chorea induced by lamotrigine. The patient was on multiple centrally active medications. Three additional cases of lamotrigine-associated chorea were identified through a literature review. In 2 of these cases, other centrally acting agents were used, and chorea was resolved with weaning lamotrigine.

**Discussion:**

Chorea is infrequently seen in the setting of lamotrigine use. In these rare cases, the presence of other centrally acting medications with lamotrigine may contribute to chorea.

**Highlights:**

Lamotrigine use is associated with movement disorders, including chorea, but the characteristics are not clearly defined. From our retrospective review, one adult had clear temporal and dose-related association between chorea and lamotrigine. We analyzed this case in conjunction with a literature review of cases of chorea associated with lamotrigine.

## Introduction

Chorea is a hyperkinetic movement disorder with multiple etiologies, including neurodegenerative disorders, brain lesions, autoimmune disorders, metabolic disorders, and certain medications [1,2,3]. Lamotrigine is a centrally acting medication that causes presynaptic blockage of type 2 voltage-gated sodium channels and high voltage-activated calcium channels, modulating the release of excitatory neurotransmitters of glutamate/aspartate and inhibitory neurotransmitter of gamma-aminobutyric acid (GABA) [4,5]. Lamotrigine has been associated with abnormal movements, including tics, myoclonus, parkinsonism, and dystonia [6,7]. In trials, chorea associated with lamotrigine therapy was noted as a rare side effect, and the onset of chorea has been reported among pediatric and adult patients treated with lamotrigine [8,9]. More recently, some studies report chorea in patients treated with a combination of antiseizure medications, such as lamotrigine and phenytoin [6]. This study aims to describe the clinical features of lamotrigine associated with the onset of chorea and potential contributing factors.

## Methods

We retrospectively reviewed the electronic health records of patients diagnosed with chorea who were evaluated at Mayo Clinic in Rochester, MN, Phoenix, AZ, and Jacksonville, FL, from 2000 to 2022. We recorded patients who developed chorea in the setting of lamotrigine use and evaluated their clinical course. Cases were excluded if chorea could be better explained by another diagnosis, such as Huntington’s disease or other degenerative or autoimmune causes, rather than an adverse effect of lamotrigine. The study was reviewed and approved by the Mayo Clinic Institutional Review Board with permission received by patients for the use of clinical information.

In addition, we performed an electronic literature search between December 2022 and January 2023. PubMed, Medline, and Scopus were searched from inception to search date to retrieve previous studies reporting chorea associated with lamotrigine use. Case reports, case series, and articles were restricted to those published in English. The search was performed using the search terms “lamotrigine associated chorea,” which extracted 64 articles, “lamotrigine induced chorea,” which extracted 61 articles, and “lamotrigine and chorea,” which extracted 274 articles. The database searches retrieved 399 total records (33 from PubMed, 337 from SCOPUS, and 29 from MEDLINE) (Supplementary Figure 1). All search results were uploaded to EndNote. 138 duplicates were removed from these records using the duplicate detection tool on EndNote. Records of 261 unique publications remained for title/abstract review. Reports associated with Huntington’s disease, Parkinson’s disease, demyelinating disease, neuroacanthocytosis syndromes, sleeping disorders, obstetrics, and movements consistent with seizures or epilepsy were excluded. Reports in the setting of genetic mutations or metabolic derangements that cause abnormal movements and manifestations of chorea associated with other medications and not including lamotrigine were also excluded. Reports of drug properties and regulation, animal experiments, and any report that did not mention chorea were excluded. Six potentially relevant cases that mentioned new-onset chorea and lamotrigine use were selected. Three additional cases were excluded because an alternative cause of chorea was deemed more likely. In addition to the patient from our institution, 3 selected cases were abstracted for patient demographics, lamotrigine dose, chorea onset and course, other medication use, neurologic history, and outcome.

## Results

A total of 591 patients were diagnosed with chorea at our institution during the study period, with 8 cases occurring in the setting of lamotrigine use. In 7 patients, other causes of chorea were considered more likely, including chorea related to antihistamine use (n = 1), Tourette’s syndrome (n = 1), viral infection (n = 1), and drug-induced parkinsonism (n = 1). In 3 patients, the onset of chorea was prior to lamotrigine initiation, making the possibility of chorea being an adverse effect of lamotrigine less likely. One patient with chorea closely associated with lamotrigine use is described below.

### Case Report

**Figure 1:**
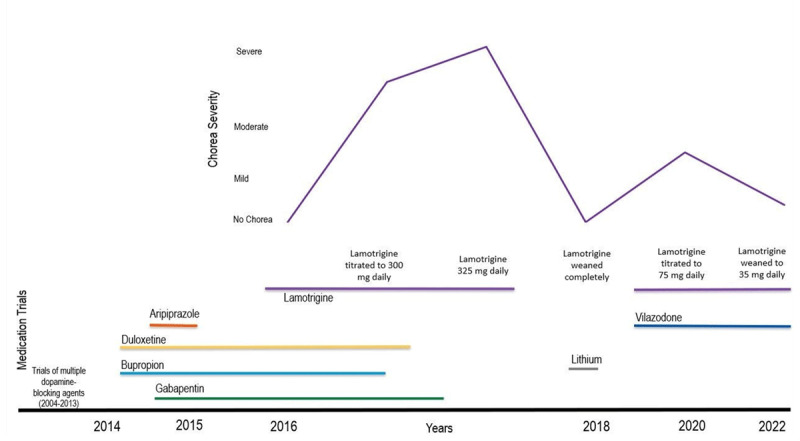
Time course of medication trials and chorea severity association. Note: timeline is for visual aid only and not to scale.

The patient is a 58-year-old right-handed woman with a history of bipolar type II disorder (depression predominant), migraine headaches, a right acoustic neuroma (schwannoma), and meningioma (surgically removed in 2013 and 2015, respectively). Other medical history is notable for cranial nerve V, VII–VIII palsy following surgical resection, and hypertension. There was a strong family history of depression but no history of chorea or other neurologic diseases. The patient had previously been treated with multiple agents for depression (Figure 1), including ziprasidone and olanzapine, for over 10 years, and trials of risperidone, aripiprazole, quetiapine, and valproic acid. These were discontinued 2 years before presentation due to the development of facial tardive dyskinesia, which later resolved. Aripiprazole, in combination with bupropion and duloxetine, was later trialed for mood stability and was discontinued over 18 months prior to the presentation for chorea because of no improvement in depression symptoms. At the dose of 300 mg nightly of lamotrigine, intermittent choreiform movements were noted by a movement disorder specialist. The movements became constant and problematic in the setting of lamotrigine (325 mg/day), bupropion (450 mg/day), duloxetine (120 mg/day), gabapentin (3200 mg/day), and trazodone (50 mg at bedtime as needed). Of note, at the time of chorea presentation, lamotrigine had been the last medication added.

On neurologic examination, there was right facial weakness (forehead, orbicularis oculi, and lower face) with complete hearing loss of the right ear and reduced sensation to pinprick over all three divisions of the right trigeminal nerve. There were choreiform movements involving the hands with a tonic inversion extension with a wrist-flexion type movement and gripping of the palm, stereotyped feet tapping and leg inversion, as well as face and truncal movements. Gait was characterized by a slightly widened base with a re-emergence of the choreiform movements. The patient felt most stable using a cane.

An extensive evaluation was performed, which included serum thyroid function tests, liver enzymes, sedimentation rate, ceruloplasmin and ferritin, vitamin B12 and folate, and a blood smear for acanthocytes, with all testing returning negative or normal. Cerebrospinal fluid (CSF) tests were performed, including the nontreponemal CSF Venereal Disease Research Laboratory Test (VDRL), which was negative, and a paraneoplastic panel. The paraneoplastic panel tested for antibodies against: ANNA-1, ANNA-2, ANNA-3, PCA-1, PCA-2, PCA-Tr, CRMP-5 IgG, AGNA-1, and Amphiphysin. Additional autoimmune labs included GABA-B R Ab, AMPA-R Ab, NMDA-R Ab, and multiple sclerosis IgG. All paraneoplastic and autoimmune labs returned negative or normal. Genetic testing for Huntington’s disease showed a single allele of 17 CAG repeats, considered most likely to be homozygous for 17 CAG repeats. This result, along with the patient’s age, suggested that this individual is very unlikely to have Huntington disease. Electromyography and movement lab evaluation showed no electrophysiological evidence for myoclonus, dystonia, orthostatic tremor, or orthostatic myoclonus. A brain MRI did not show any change in the basal ganglia or evidence of caudate atrophy. Medication-induced chorea was suspected. Bupropion, duloxetine, and gabapentin were rapidly tapered in the respective order (Figure 1) and further discontinued over weeks. Chorea decreased in severity; however, it did not resolve, and the patient continued to have involuntary movement affecting extremities. Chorea associated with lamotrigine use was suspected, so lamotrigine was weaned entirely. Lithium was trialed for 6 weeks for mood stability but discontinued due to worsening depression, and vilazodone was begun as a mood stabilizer agent. Chorea markedly improved within 2–3 months of lamotrigine discontinuation to the point of disappearance; however, depression symptoms continued to recur. After a thorough discussion with the patient on the risks/benefits of lamotrigine, the medication was restarted six months later. At the dose of 75 mg/day, chorea reappeared with bothersome severity. Different doses of lamotrigine were trialed with the dose of 35 mg/day, leading to mood stability with mild chorea tolerable to the patient. The patient has been stable on this regimen for 2 years.

### Literature review

Our search yielded 261 unique articles, of which 3 could clearly associate chorea with the use of lamotrigine (Table 1). Among patients reported in the literature, the median age at the time of chorea diagnosis was 23 (range 8–52). All patients were male. Two patients were adults, and one was pediatric. Two patients were using lamotrigine to treat epilepsy. One patient was using lamotrigine for psychiatric disease and was found to have suspected chorea after an acute lamotrigine overdose. Following lamotrigine administration, two patients experienced acute chorea onset (0–4 days), and one patient had an unclear onset. Long-term outcomes included improvement of chorea with lamotrigine discontinuation in two cases. The long-term outcome for the patient that overdosed on lamotrigine was not included in the literature. However, chorea was reported to subside 12 hours after the subsequent overdose.

**Table 1:** Pooled Abstracts/Characteristics of Lamotrigine Associated Chorea.


CASE	AGE/GENDER	LAMOTRIGINE DOSE	CHOREA ONSET	CHOREA RESOLVED	OTHER MEDICATIONS	ADDITIONAL NEUROLOGIC HISTORY	FOLLOW-UP/OUTCOME

Das KB, et al., 2003 [9]	8/M	8 mg/kg/d	Unclear	3 wk	Valproate ‡	Seizures	Asymptomatic after 4 years

Zesiewicz TA, et al., 2006 [3]	52/M	100 mg/d	2 d	2 wk	Phenytoin ^§^ Citalopram	Seizures DepressionNarcolepsy	NR

Miller MA, et al., 2008	23/M	Level 63.9 µg/mL *	2 hr †	12 hr	║	Bipolar disorder	NR

**New case**	58/F	325 mg/d	Unclear	~ 2–3 mo.	Bupropion Duloxetine Gabapentin Trazadone	Schwannoma #, Meningioma #, migraine HA, Bipolar disorder, CN palsy **	Lamotrigine restarted (lower dose) Mild chorea present


d indicates day; wk-weeks; hr- hours, mo.- months; NR- not reported; HA- headache; CN- cranial nerve. * Level obtained 4 hours after admission; † Indicates hours after subsequent overdose. ‡ Valproate weaned, patient on lamotrigine monotherapy at time of chorea onset. § Phenytoin discontinued several weeks prior to starting lamotrigine and chorea onset. ║ Additional medications not reported; # Surgically removed; ** CN V, VII, VIII palsy.

## Discussion

Chorea associated with lamotrigine use is rare. In review of all cases of chorea at our institution, only one patient presented with chorea that was closely linked with lamotrigine. In this case, titration of lamotrigine clearly affected the severity of chorea. The literature review showed 3 additional cases of patients with lamotrigine-associated chorea [3,9,10].

There are interesting features to note in evaluating the 4 cases. First, both children and adults were affected. In children, movement disorders are associated with anti-seizure medications in the setting of an underlying structural lesion [11]. While our patient had intracranial masses (acoustic neuroma and meningioma), both were resected prior to the onset of chorea. One possible unifying feature in children and adults is the use of other centrally acting medications in addition to lamotrigine. In the case we report, and in 2 of the 3 cases from the literature, patients were on additional antidepressant and anticonvulsant medications. These medications included: citalopram, duloxetine, bupropion, phenytoin, sodium valproate, gabapentin, and trazodone. The combination of centrally acting agents has been postulated to contribute to the appearance of chorea [7]. For example, some papers report that using phenytoin with other medications causes an increase in free phenytoin levels and thus increases the risk of abnormal movements [12].

Lamotrigine is used for several indications, including focal seizures, generalized seizures, and maintenance of bipolar type I disorder. Off-label uses include bipolar depression, fibromyalgia, and pain [4,13]. The mechanism of action for lamotrigine is not entirely understood. The main actions involve presynaptic blockage of type 2 voltage-gated sodium channels and high-voltage-activated calcium channels. This modulates the release of excitatory neurotransmitters of glutamate/aspartate and inhibitory transmitters of gamma-aminobutyric acid (GABA) [4]. The decrease of glutamate/aspartate increases the firing of serotonergic neurons [14], which may account for the effects of lamotrigine on mood stabilization. Lamotrigine’s effects on GABA are complex and vary based on the site of action [5]. In one study, lamotrigine was found to reduce glucose metabolism in the thalamus, basal ganglia, and multiple regions of the cerebral cortex [15]. Although no one study has fully explained the occurrence of chorea, it has been hypothesized that partial reduction of tonic globus pallidus internus (GPi) activity could cause target neurons in the thalamus to depolarize near threshold so that random, small fluctuations in other, non-basal ganglia inputs can cause them to fire in random bursts [16]. It is possible that the effect of lamotrigine on glucose metabolism in the thalamus, basal ganglia, and cerebral cortex could affect the depolarization in the thalamus and thus lead to chorea. This is speculation, though, and the pathophysiology of chorea onset in the setting of lamotrigine requires further investigation. It should also be recognized that other hyperkinetic disorders have been linked to lamotrigine use, including but not limited to tremors, tics, and dystonia [6].

There are limitations to this study. First, it is a single-institution, retrospective study. The number of cases is small, which makes drawing inferences problematic. While we made all attempts to capture all published cases of chorea in the setting of lamotrigine, there are limitations of the literature database search engine, including subject headings used to identify chorea in the setting of lamotrigine such that it is possible that we did not capture all cases. Additionally, most patients were taking multiple medications, and other factors could have contributed to the onset of chorea. As lamotrigine is used for many different patients and disorders, knowledge of potential side effects is important.

Chorea is a rare adverse effect of lamotrigine use that can present in both children and adults. Concurrent use of other centrally acting medications, in addition to lamotrigine and higher doses of lamotrigine, may contribute to the onset of chorea in these patients.

**Figure:**
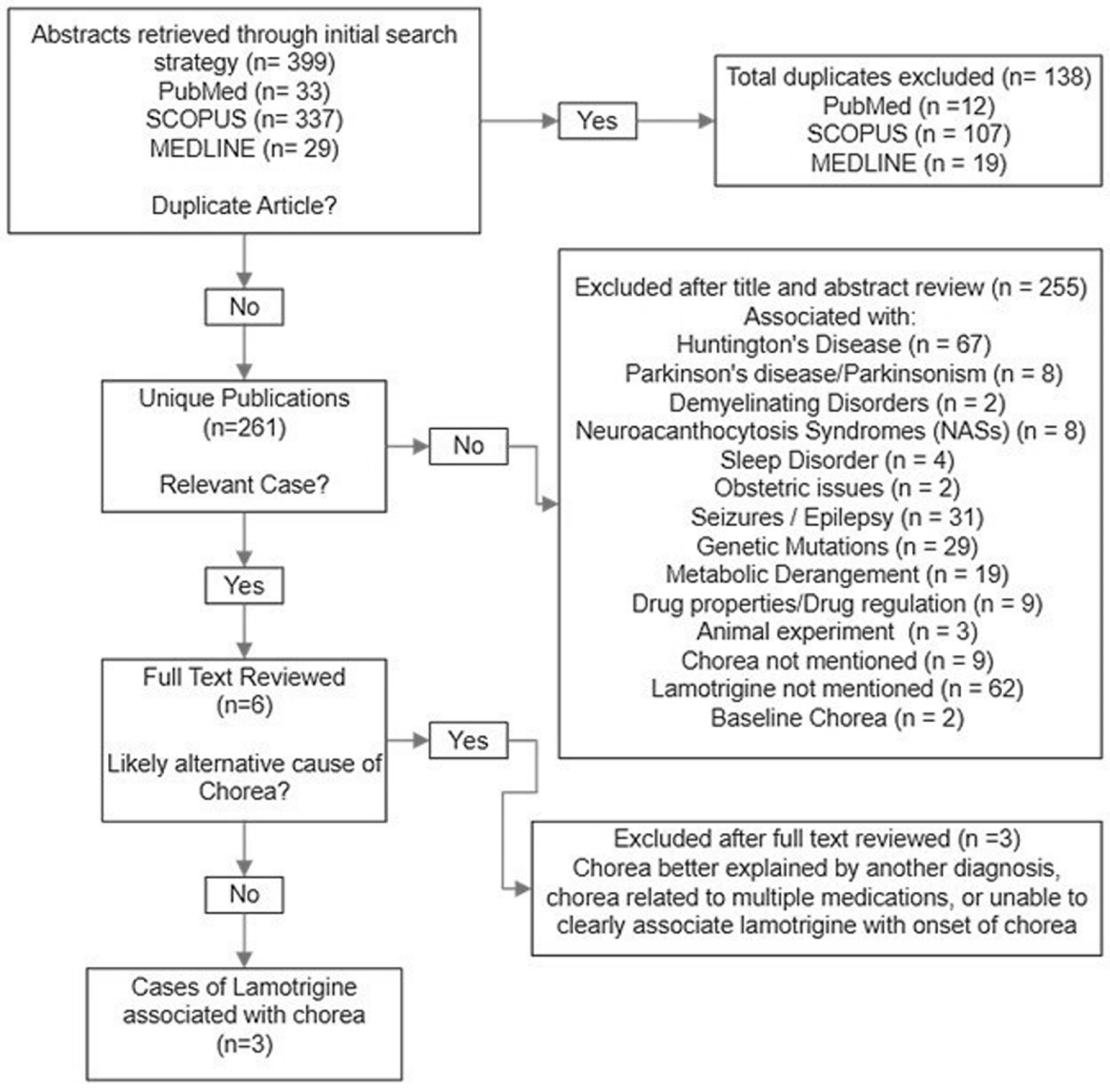
**Supplementary Figure 1 Flow Diagram of Literature Case Abstraction.** The following steps were taken to review the cases of lamotrigine-associated chorea in the literature.
